# In Silico and In Vitro Studies of Novel Azomethines on DNA Repair Genes in Gastric Cell Lines

**DOI:** 10.3390/life13101982

**Published:** 2023-09-28

**Authors:** Alpaslan Ozturk, Tugba Agbektas, Alakbar Huseynzada, Ruslan Guliyev, Rana Ganbarova, Ulviyya Hasanova, Ayca Tas, Sultan Erkan, Cemile Zontul, Nihal Inandiklioglu, Yavuz Silig

**Affiliations:** 1 Clinical Biochemistry, Etlik City Hospital, 06170 Ankara, Turkey; 2 Department of Food Processing Technologies Services, Yıldızeli Vocational School, 58500 Sivas, Turkey; tubaagbektas@cumhuriyet.edu.tr; 3 Industrial Chemistry Research Laboratory, Baku State University, Z. Khalilov 33, Baku AZ1148, Azerbaijan; alakbar.huseynzada1117@gmail.com (A.H.); ruslanguliyev@gmail.com (R.G.); ranaganbarova@gmail.com (R.G.); ulviyyahasanova@gmail.com (U.H.); 4 GPOGC SRI, Azerbaijan State Oil and Industry University, Baku AZ1010, Azerbaijan; 5 Department of Chemistry, Azerbaijan Engineers Union, Bashir Safaroglu 118, Baku AZ1022, Azerbaijan; 6 ICESCO Biomedical Materials Department, Baku State University, Z. Khalilov 33, Baku AZ1148, Azerbaijan; 7 Department of Nutrition and Diet, Faculty of Health Sciences, Sivas Cumhuriyet University, 58140 Sivas, Turkey; aycatas@cumhuriyet.edu.tr; 8 Department of Chemistry, Faculty of Science, Sivas Cumhuriyet University, 58140 Sivas, Turkey; sultanerkan@cumhuriyet.edu.tr; 9 Department of Chemistry and Chemical Processing Technologies Services, Yıldızeli Vocational School, 58500 Sivas, Turkey; cemilezontul@cumhuriyet.edu.tr; 10 Department of Medical Biology, Faculty of Medicine, Yozgat Bozok University, 66100 Yozgat, Turkey; nihal.inandiklioglu@yobu.edu.tr; 11 Department of Biochemistry, Faculty of Medicine, Sivas Cumhuriyet University, 58140 Sivas, Turkey; ysilig@cumhuriyet.edu.tr

**Keywords:** azomethine group, cytotoxicity, DNA repair gene, gastric cancer, gene expression, molecular docking

## Abstract

We herein report the determination of the cytotoxic activity and expression profiles of some DNA repair genes of newly synthesized azomethines in the gastric cancer cell line (AGS). The studied novel compounds were synthesized by a condensation reaction and received compounds were characterized by ^1^H and ^13^C NMR spectroscopy methods. Furthermore, they were applied to the AGS cell line at eight different concentrations (0.1–50 µg/mL). Anticancer activities were determined using the MTT method. Expression levels of *ATR*, *ERCC1*, *TOP2A*, and *ABCB1* genes were determined by the RT-PCR method. Biochemical parameters were also examined. The interaction of proteins with other proteins was investigated with the String v11 program. The IC50 values of compounds **1**, **2**, and **3** obtained after 72 h were 23.10, 8.93, and 1.58 µg/mL, respectively. The results demonstrate that the cytotoxic activity of compound **3** on AGS cancer cells is higher in comparison with other molecules. It was determined that the expression levels of *ATR*, *TOP2A*, and *ABCB1* genes in compounds **1**, **2**, and **3** were decreased compared to the control group. In addition, it was determined that *ERCC1* gene expression increased in compound **3**, decreased in compound **2**, and remained unchanged in compound **1** (*p* < 0.001). In AGS gastric cancer cells, a 64% decrease was detected for GST levels in compound **1**, while a 38% decrease in GSH levels in compound **2**. In addition, compounds **1–3** were examined at the molecular level with computational techniques and the docking studies revealed 4LN0 as a target protein.

## 1. Introduction

Gastric cancer is a disease that is common all over the world and is important for public health. Causes of stomach cancer include diet, smoking, alcohol, environmental factors, and Helicobacter pylori infection [1]. Helicobacter pylori, a Gram-negative microaerophilic spiral bacterium found in the gastric mucosa in patients with severe gastritis and chronic atrophic gastritis, has been accepted as an important risk factor for gastric cancer [2,3]. Gastric cancer development is a multi-step process that causes numerous genetic and epigenetic changes in oncogenes, tumor suppressor genes, DNA repair genes, cell cycle regulators, and signaling molecules [4,5].

Factors of exogenous or endogenous origin cause DNA damage and thus DNA repair mechanisms are activated. There are different types of cellular DNA repair pathways responsible for repairing DNA damage, such as base damage (BER), DNA single-strand breaks (single-strand break repairs (SSBRs)), DNA double-strand breaks (homologous recombination (HR) and non-homologous end-joining repair (NHEJ)), bulky lesions (nucleotide excision repair (specialized DNA repair systems have been developed to identify and repair NER)), and the mismatch repair (MMR) of mismatched bases [6]. Genomic instability resulting from defects in DNA repair mechanisms leads to various cancers and diseases. Damage to DNA is corrected by DNA repair mechanisms, thus preserving the genomic integrity of the cells. With these mechanisms, the recognition of the damage occurs in three steps by removing the damaged part and filling the gap. DNA damage occurs regardless of replication or through different pathways during replication [7,8,9]. Interindividual differences in DNA repair mechanisms may also affect the initiation and progression of cancer and thus prognosis. It has been shown that the BER pathway has an important role in repairing endogenous and exogenous base damage. Changes in DNA repair and gene expression levels in the BER pathway can alter the DNA repair capacity. This may affect the progression of cancer and clinical responses, such as chemotherapy [10]. Chemotherapy may be an option to improve the prognosis in advanced patients; however, the administration of drugs that are toxic to cancer cells stops the mechanism responsible for dividing cells. In this treatment method, healthy cells are damaged and if the cancer is at an advanced stage, it results in a low success rate [11].

Recently, researchers have gained knowledge of the anticancer effects of newly synthesized molecules containing the azomethine group. Drugs containing the azomethine group are among the new drugs synthesized with stronger and more selective properties and may be promising for cancer treatment (Table 1). Azo-azomethines are organic dyes and contain characteristic chromophore groups -N=N- and -CH=N-. Azo colors include amido-azo, oxy-azo, diazo, tetrazo, and other polyazo compounds. It has been noted that some azo dyes present strong antimicrobial activities [12]. These compounds and their complexes show many biological activities, including anti-tumor, antibacterial, fungicidal, and anticarcinogenic [13,14].

Taking into account the above-mentioned ideas, in the present study, we aim to investigate the in vitro and in silico anticancer activities of azomethine-containing compounds on DNA repair (*ATR*, *ERCC1*, *TOP2A*, and *ABCB1*(*MDR1*)) genes.

## 2. Materials and Methods

### 2.1. Synthesis Procedure

Compounds were synthesized by dissolving 0.1 gr of subsequent aldehyde in 5 mL of acetonitrile, followed by the addition of 0.1 mL of subsequent amine and stirring at room temperature for 2 h. Then, the reaction mixture was poured on ice and forming precipitate was filtered, washed with distilled water, and dried. The structure of the synthesized compounds was determined by NMR spectroscopy.

1H NMR spectrum of compound **1**: (CDCl3, δ, ppm), 2.84 t (6H, 6NCH2), 3.54 t (6H, 6NCH2), 6.16 s (3H, 3Ar), 6.94 s (3H, 3Ar), 7.89 s (3H, 3CHN), and 14.45 s (3H, 3OH). 13C NMR spectrum of compound **1**: (CDCl3, δ, ppm), 55.29 (3NCH2), 55.80 (3NCH2), 56.18 (3OCH3), 108.71 (3C, Ar), 116.87 (3CH, Ar), 118.04 (3C, Ar), 124.55 (3CH, Ar), 149.32 (3C, Ar), 153.29 (3C, Ar), and 165.59 (3NCH).

1H NMR spectrum of compound **2**: (DMSO-d6, δ, ppm), 3.35–3.79 m (18H, 2OCH3 + 6OCH2), 7.33 s (2H, Ar), 8.05 s (2H, Ar), 8.64 s (2H, 2CH=N), and 12.75 s (2H, 2OH). 13C NMR spectrum of compound **2**: (DMSO-d6, δ, ppm), 51.28 (2NCH2), 55.89 (2OCH3), 68.82 (2OCH2), 70.16 (2OCH2), 105.85 (2CH, Ar), 111.7 (2CH, Ar), 126.05 (2C, Ar), 132.49 (2C, Ar), 152.32 (2C, Ar), 167.89 (2C, Ar), and 173.09 (2CH=N).

1H NMR spectrum of compound **3**: (DMSO-d6, δ, ppm), 2.79 t (6H, 6NCH2), 3.51 t (6H, 6NCH2), 6.29 s (3H, 3Ar), 6.84 s (3H, 3Ar), 8.09 s (3H, 3CHN), and 14.11 s (3H, 3OH). 13C NMR spectrum of compound **3**: (DMSO-d6, δ, ppm), 58.29 (3NCH2), 60.80 (3NCH2), 61.18 (3OCH3), 106.71 (3C, Ar), 120.84 (3CH, Ar), 119.11 (3C, Ar), 125.59 (3CH, Ar), 144.39 (3C, Ar), 157.22 (3C, Ar), and 169.51 (3NCH).

### 2.2. In Silico Calculations

The synthesized molecules were calculated using Gauss software by the hybrid density functional theory (DFT) method [15,16]. Compounds were optimized at the B3LYP/6-31+G(d,p) level. Quantum chemical parameters, such as the highest occupied molecular orbital (HOMO), the lowest unoccupied molecular orbital (LUMO), and the energy difference between LUMO and HOMO (ΔE), frontier molecular orbitals, and molecular electrostatic potential (MEP) map contour graphics, were obtained from the optimized structures. Molecular docking calculations were performed using Maestro 12.8. Both ligands and target proteins were minimized by the OPLS4 method in the molecular insertion calculations. The target protein represented the gastric cancer cell line (PDB ID: 4LN0) [17].

### 2.3. Cell Culture

AGS gastric cancer cells were grown in Dulbecco’s Modified Eagle’s Medium (DMEM) containing 100 Units/mL of penicillin and 10% fetal bovine serum (FBS). This was incubated at 37 °C under 5% CO_2_ oven conditions. The cell line was passaged after a certain growth rate.

### 2.4. In Vitro Cytotoxicity Determination (MTT)

After AGS gastric cancer cells adhered to 75 fluxes, 2 mL of Trypsin/EDTA was added and allowed to incubate again in an oven with 5% CO_2_, so the cells were separated from the flux surface. The AGS cell line was seeded in 96-well plates at a ratio of 1 × 10^5^ cells/well and different compound concentrations (0.1–50 µg/mL) were dosed to incubate for 24, 48, and 72 h. Cells with only DMSO and no components applied were used as the controls to determine cell viability. Only a cell culture medium was added to the blank wells. Accordingly, the background correction was created. The cytotoxicity of the compounds on the AGS cell line was determined by the MTT method (3-(4,5-dimethyl thiazol-2-yl)-2,5-diphenyl tetrazolium bromide). A total of 10 μL was added to the well and incubated. MTT and medium were aspirated and 100 μL of DMSO was added to each well and left on a stirrer for 15 min at room temperature. The entire experimental procedure was performed in triplicate. After this period, absorbance at 570 nm was measured using the GraphPad Prism7 program and the IC_50_ values were determined. The absorbance measurements obtained from the control and experimental groups to which the components were applied were averaged. Standard deviation values were calculated. The percentage of cell viability was obtained by comparison with the control according to the following equation: Cell viability = (experimental group OD − blank group OD)/(DMSO solvent control group OD − blank group OD) × 100% (OD = optical density). Accordingly, the potential of the applied components to inhibit cell growth was evaluated.

### 2.5. Cell Morphology

AGS gastric cancer cells (5 × 10^5^ cells/well) were plated on plates. Compounds **1**, **2**, and **3** were dosed at 1 µg/mL to each cell in the wells. Changes in cell morphology were observed with a 20× magnification (Axio Vert.A1, Zeiss, Oberkochen, Germany) cell imaging device.

### 2.6. Bioinformatics Analysis

The interactions of ATR, ERCC1, TOP2A, and ABCB1 (MDR1) proteins were performed with the STRING program available at https://string-db.org/ (accessed on 27 July 2023).

### 2.7. RNA Isolation from Cell Culture Samples

The IC_50_ concentrations of each sample were determined after 48 h of incubation, and AGS cells that reached a certain growth rate were seeded in 6-well plates. Compounds were dosed after 24 h of incubation. Then, RNA isolation from the AGS cell line was performed with the RNeasy Plus Mini kit protocol.

### 2.8. cDNA Synthesis

The obtained RNAs were synthesized as cDNA in accordance with the cDNA synthesis kit protocol.

### 2.9. Real-Time PCR Analysis

The expression levels of *ATR*, *ERCC1*, *TOP2A*, and *ABCB1 (MDR1)* genes were determined in the RT-PCR device with the optimized RT^2^ SYBRGreen qPCR Mastermix kit. SYBRGreen was used as a fluorescent dye and 25 µL of qPCR mixture was prepared from the cDNA-containing samples according to the kit protocol. The statistical analysis of the data was performed using the ΔΔCT method with https://dataanalysis2.qiagen.com/pcr software (accessed on 27 July 2023).

### 2.10. Determination of Antioxidant Levels

#### 2.10.1. Determination of Glutathione (GSH)

GSH is an important intracellular antioxidant that acts as a regulator of cellular redox by protecting cells from damage caused by lipid peroxides, reactive oxygen, and nitrogen species, as well as xenobiotics. Compounds **1**, **2**, and **3** were dosed with this method after the incubation of AGS cells in 6-well plates. After 48 h of incubation, 250 µL of cell medium was obtained and analyzed according to the protocol of Giustarini et al. [18].

#### 2.10.2. Determination of Glutathione-S-Transferase (GST)

GST enzymes perform the detoxification process by providing conjugation to various electrophilic compounds, and thus the damage that may occur due to oxidative stress is prevented. In this method, 250 μL of samples incubated with AGS cells were obtained and analyzed according to the protocol of Ghelfi et al. [19].

#### 2.10.3. Catalase Determination (CAT)

CAT activities, viz., the detoxification ability in some tumors, are important in terms of CAT levels. In this method, 100 µL of AGS cell medium incubated for 48 h was obtained and analyzed in accordance with the protocol of Aebi et al. [20].

### 2.11. Membrane Integrity

#### Determination of Lactate Dehydrogenase (LDH)

This method was used to measure LDH levels for detecting anticancer activity and to determine the activity of cytoplasmic enzymes released from damaged cells. The cell cytotoxicity of AGS treated with compounds **1**, **2**, and **3** was determined by the measurement of LDH enzyme activity. A total of 100 µL of cell culture medium was obtained and the LDH determination was performed in accordance with the protocol of Decker et al. [21].

## 3. Results

### 3.1. Synthesis Part

The synthesis of the targeted compounds was performed by the simple condensation of aldehydes with the subsequent amines. The advantageous side of the synthesis was that there was no need for additional purification stages for the obtained during the synthesis precipitates, only washing with a small amount of distilled water was enough. Another positive side was that the reactions proceeded in a non-catalytic medium. The formed precipitates were analyzed by ^1^H and ^13^C NMR methods. The disappearance of the aldehyde group peak and the presence of the azomethine bond peak in the NMR spectra proved the formation of the investigated compounds.

### 3.2. In Silico Studies

#### Frontier Molecular Orbitals (FMOs) and MEP Contours

**Figure 1:**
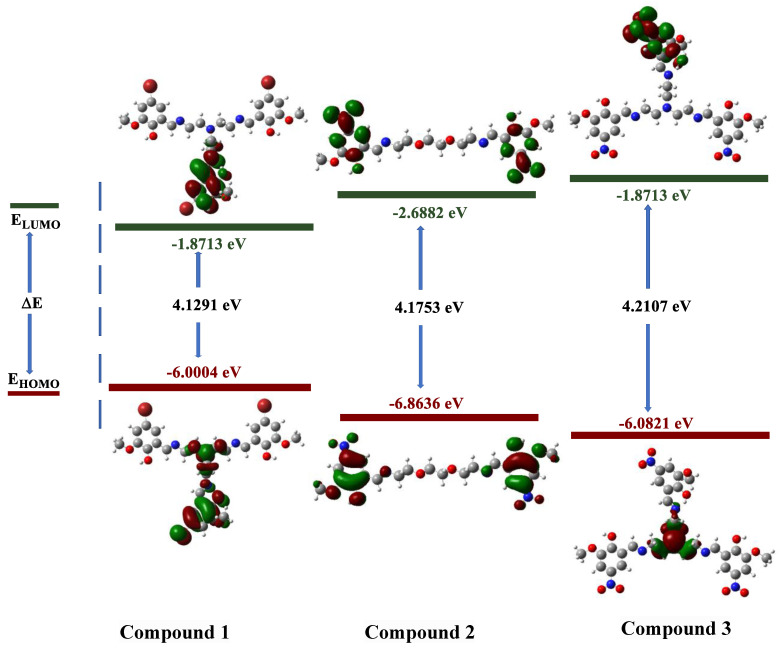
Contour diagram of the frontier molecular orbitals of compounds **1**–**3**.

The electronic properties of synthesized molecules can be elucidated by DFT methods. The determination of electron acceptor and donor groups is important for the compounds examined at the molecular level in drug studies. The interaction regions of the molecules and the numerical strength of the electron densities of the functional groups in these regions can make the interaction with biological receptors more understandable. For this purpose, frontier molecular orbitals (HOMOs and LUMOs), which were important in the electron exchange of compounds **1**–**3**, were analyzed. Contour diagrams of the electron distribution in these orbitals and their energies are presented in Figure 1.

The electron distributions of the frontier orbitals demonstrate that the ground-state electron density (HOMO) is on the phenyl rings. It can be observed that the electron is still on the phenyl rings in case of excitation or transition to a higher energy state (LUMO). This reveals that electron exchange occurs over π molecular orbitals [22,23].

**Figure 2:**
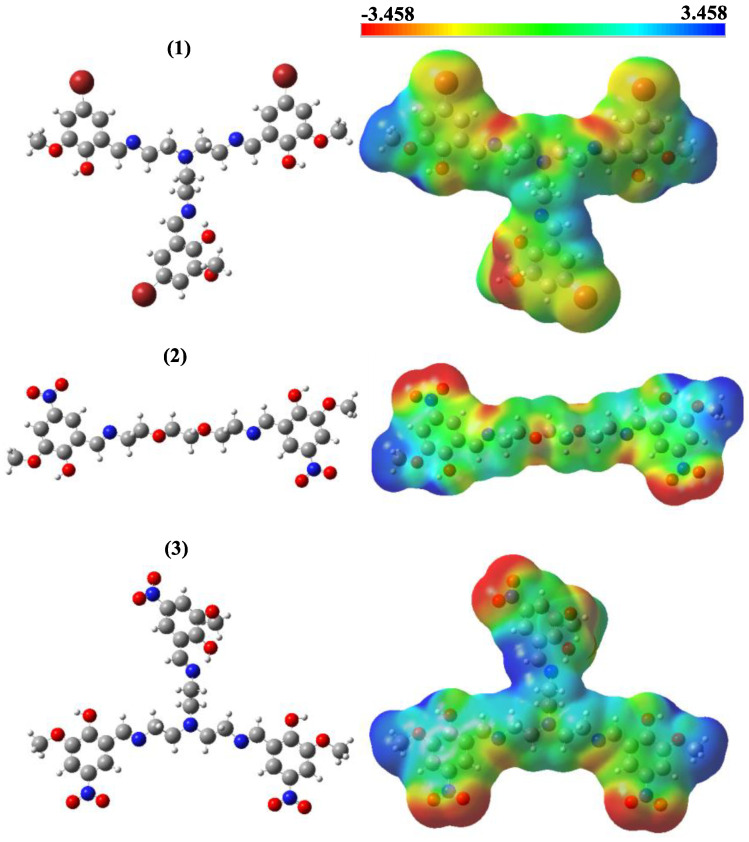
Optimized structures and molecular electrostatic potential maps of molecules **1**, **2** and **3**.

When our compounds were examined, it was observed that OH and CH=N substituents were more likely to interact with biological receptors. Compounds **1**–**3** presented in Figure 1 were optimized with the B3LYP/6-31+G(d,p) level, and the quantum chemical parameters, such as E_HOMO_, E_LUMO_, and ΔE, were calculated for the synthesized compounds. E_HOMO_, E_LUMO_, and ΔE are very important to explain the quantum chemical properties of the studied compounds, due to the fact that there is an undeniable relationship between quantum chemical parameters and biological activity. The HOMO energies of compounds **1**–**3** were −6.0004, −6.8636, and −6.9800 eV, respectively. The LUMO energies were −1.8713, −2.6882, and −2.7571 eV, respectively. The ΔE values were 4.1291, 4.1753, and 4.2230 eV, respectively. The increasing value of HOMO energy and decreasing value of LUMO energy and ΔE positively affected the biological activity. Therefore, the obtained results indicate that compound **3** exhibits more activity, whereas compound **1** exhibits less activity. In addition, molecular electrostatic potential (MEP) maps allow the electronic behavior of compounds to be analyzed at the atomic scale. In other words, the electrophilic and nucleophilic attack sites of molecules can be detected. The optimized structures and MEP maps of the synthesized compounds with the calculation at B3LYP/6-31+G(d,p) level are presented in Figure 2. The red color in the MEP map indicates the electron-rich region, while the dark blue indicates the electron-poor region. Specifically, the -OH and -CH=N substituents are the sites for nucleophilic attacks, which increase the reactivity of **1**–**3**.

### 3.3. Molecular Docking

Molecular docking studies were performed to investigate the anticancer activity of the studied compounds against gastric cancer. The selected target protein (PDB ID: 4LN0) was the Hippo pathway of gastric cancer. The calculated docking score (DS), van der Walls energy (EvdW), Coulomb interaction energy (ECoul), and total interaction energy (ETotal) are presented in Table 2.

**Figure 3:**
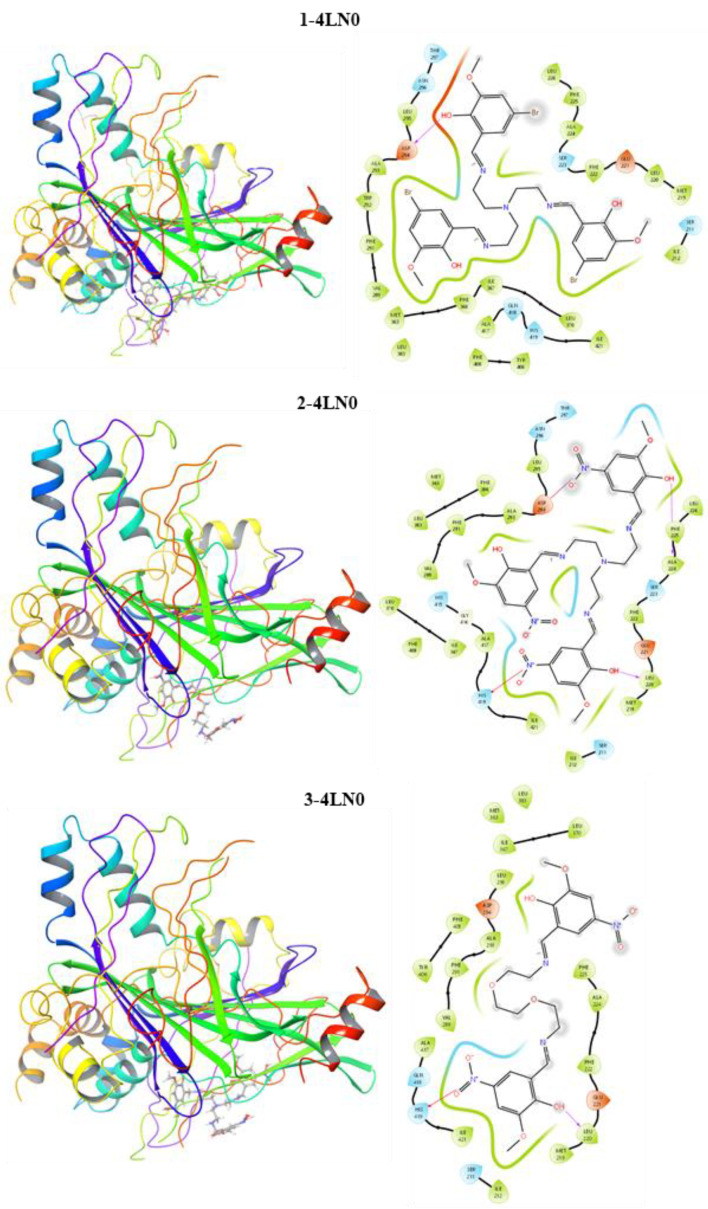
Interaction maps of compounds **1**, **2** and **3** with the Hippo pathway of gastric cancer.

The docking score is an empirical measure used to distinguish the binding ability between the studied ligands and the protein [24]. E_vdW_ indicates the ligand–protein interaction strength of secondary chemical interactions. E_Coul_ and E_Total_ show the key–lock system relationship between the ligand–protein [25]. The docking results are in agreement with the experimental data. Compound **3** has a docking score higher than the other compounds and Docetaxel reference. Compound **1** is more active than the reference substance; however, it exhibits lower activity than the other synthetic compounds. These results are in good agreement with the DFT calculations. Additionally, the interaction maps for the ligands and target proteins are represented in Figure 3, which indicates the mode of binding between the ligands and target protein. It is thought that hydrogen bonding, which is one of the interaction types, plays an active role between the ligand and receptor. Compounds **3** and **2** were found to form H-bonds with the leucine amino acid residue. Compound **1** formed an H-bond with the aspartic amino acid residue. In addition, within the interaction types, hydroxyl groups exhibited polar and hydrophobic interactions.

### 3.4. In Vitro Assay for Cytotoxicity Activity (MTT Assay)

**Figure 4:**
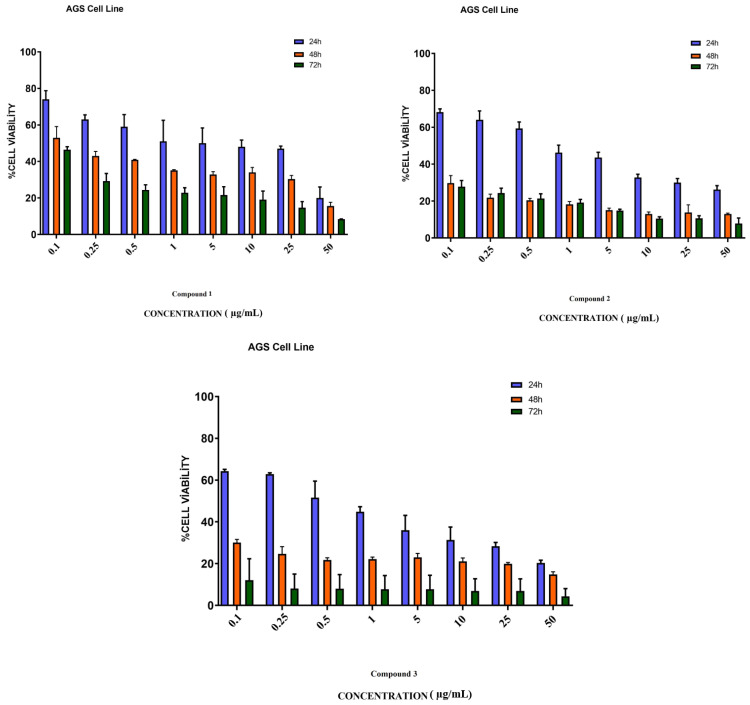
Cytotoxicity study of compounds **1**, **2**, and **3** in AGS cells. AGS cells were treated with studied molecules for 24, 48, and 72 h in a concentration range of 0.1 to 50 µg/mL. This figure shows the mean ± SEM values of three separate experiments.

Eight different concentrations of compounds **1**, **2**, and **3**, which were applied to AGS gastric cancer cells, were dosed in the range of 0.1–50 µg/mL. As a result of the analysis of the MTT method used in the determination of the cell viability of the compounds, the cytotoxic activities of the compound-administered cancer cells and the non-compounded control groups were compared and their cytotoxic activities were determined. As a result of this analysis, the cytotoxic activities of the compounds applied to the AGS gastric cancer line were most active after 72 h of incubation. The IC_50_ values of compounds **1**, **2**, and **3** obtained after 72 h were 23.10, 8.93, and 1.58 µg/mL, respectively. When the results were evaluated, the cytotoxic activity of compound **3** on AGS cancer cells was found to be more active than other molecules (Figure 4).

### 3.5. Cell Morphology Analysis

**Figure 5:**
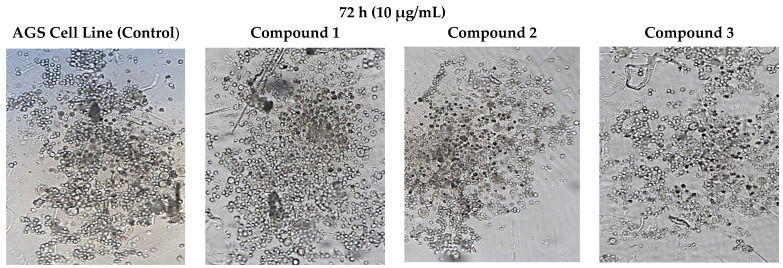
Morphological changes in AGS cells after 72 h of incubation with concentrations (10 µg/mL) of compounds **1**, **2**, and **3**. The presented results were achieved microscopically.

As a result of the application of compounds **1**, **2**, and **3** to each AGS cancer cell in the wells at a dose of 1 µg/mL, the cancer cell morphology changed in the morphological determinations compared to the control group (Figure 5).

### 3.6. Bioinformatics Analysis

**Figure 6:**
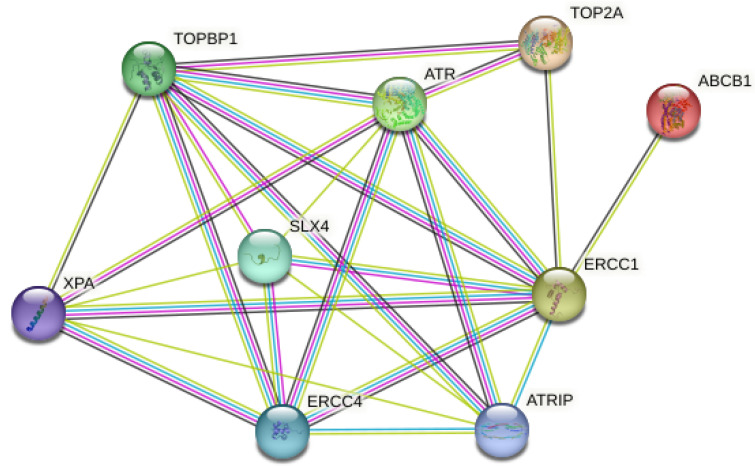
Schematic representation of known and predicted protein–protein interactions with ATR, ERCC1, TOP2A, and ABCB1 (MDR1) proteins in the STRING database.

As a result of the analysis of the expression levels of ATR, ERCC1, TOP2A, and ABCB1 (MDR1) genes on AGS gastric cancer detected by the RT-PCR method, the multi-protein STRING network analysis was applied to determine the functional interactions of the proteins formed as a result of these genes in cellular processes. Protein–protein interactions of the proteins involved in the DNA repair mechanism were investigated. These proteins were confirmed to be associated with TOPBP1, ATRIP, SLX4, ERCC4, and XPA proteins (Figure 6). Protein–protein interactions with 9 proteins in the first shell (ATR, ERCC1, TOP2A and ABCB1 (MDR1), TOPBP1, ATRIP, SLX4, ERCC4, and XPA) were in the range of a 0.999–0.450 homology score and were statistically significant (*p* < 0.05). There were protein–protein interactions with 25 proteins at the second shell level (Figure 6) (Table 3). The PPI enrichment *p*-value was 1.72 × 10^−8^. In light of these results, it is of great importance to investigate other related proteins in future studies.

### 3.7. Gene Expression Analysis

**Figure 7:**
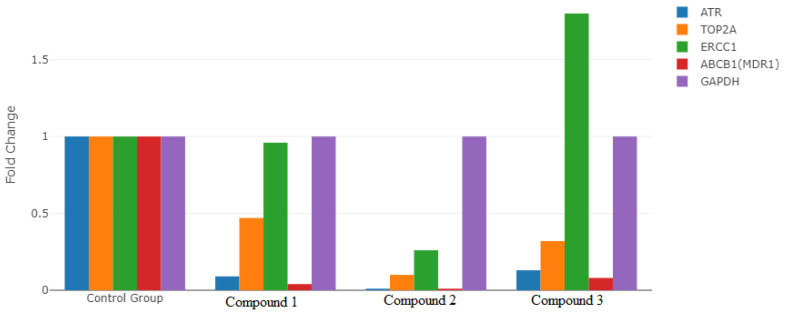
Comparison of the expression levels of *ATR*, *ERCC1*, *TOP2A*, and *ABCB1 (MDR1)* genes in the groups.

In our study, the *GAPDH* gene was used as the reference gene applied to the AGS gastric cancer cell line. RT2 profiler RT-PCR Sequence Data Analysis version 3.5 software with Rotor-Gene 6000 software was applied in the statistical analysis of the RT-PCR results of the analyzed genes. The rates of change in *ATR*, *ERCC1*, *TOP2A*, and *ABCB1 (MDR1)* gene expression levels of the compounds used in the present study are presented in Figure 7. It was determined that the expression levels of *ATR*, *TOP2A*, and *ABCB1 (MDR1)* genes in compounds **1**, **2**, and **3** decreased compared to the reference gene, *GAPDH*. In addition, it was determined that *ERCC1* gene expression increased in compound **3**, decreased in compound **2**, and remained unchanged in compound **1**, compared to the control group. Statistically significant differences were observed between the study groups in the expression levels of all the genes evaluated in our study (*p* < 0.001) (Figure 7) (Table 4).

### 3.8. Determination of Antioxidant Levels

#### 3.8.1. Glutathione Determination (GSH)

**Figure 8:**
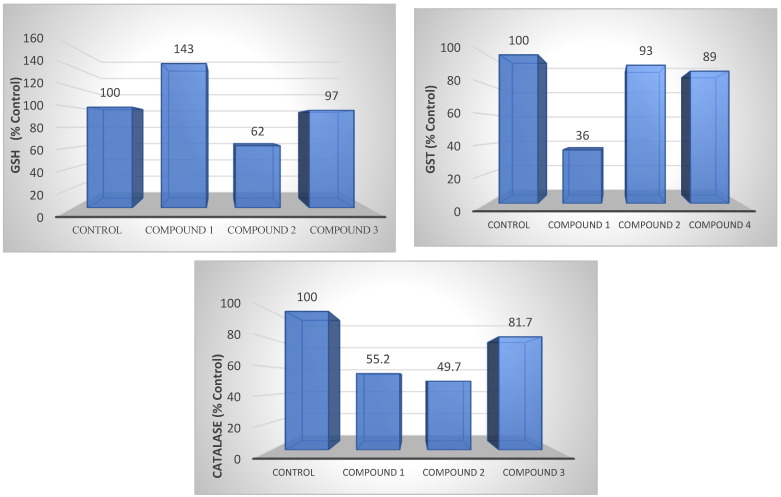
GSH, GST, and CAT levels of compounds **1**, **2**, and **3** in AGS cell lines. The determination of GSH, GST, and CAT levels also shows the antioxidant level.

Compared to the control group, GSH levels increased by 43% in compound **1** and decreased by 38% in compound **2** in AGS gastric cancer cells (Figure 8).

#### 3.8.2. Glutathione-S-Transferase Determination (GST)

Compound **1** presented a 64% decrease in GST levels in AGS gastric cancer cells, while no change was observed in the other groups (Figure 8).

#### 3.8.3. Catalase Determination (CAT)

Compounds **1** and **2** presented a 50% reduction in CAT levels in AGS gastric cancer cells compared to the control group, while there was a slight decrease in compound **3** (Figure 8).

### 3.9. Membrane Integrity

#### Lactate Dehydrogenase Determination

**Figure 9:**
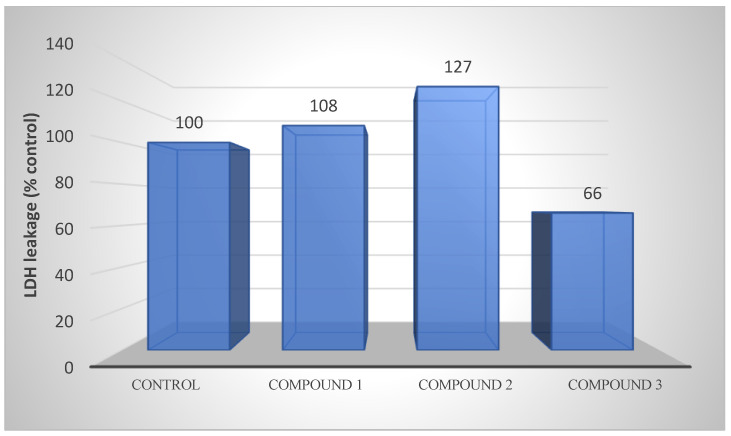
LDH releases of compounds **1**, **2**, and **3** from AGS cells after 48 h of incubation at the IC50 concentration. LDH activity is also an indicator of membrane integrity.

LDH levels decreased by 40% in compound **3** in AGS gastric cancer cells, while a slight increase was observed in other compounds (Figure 9).

## 4. Discussion

In our study, we detected the anticancer activity of azomethine-containing compounds **1**, **2**, and **3** on the AGS gastric cancer cell line and investigated how this activity had an effect on the function of DNA repair genes. As a result, it was determined that these compounds had anticancer effects after 72 h of incubation, which was the most active on AGS cell lines. Then, the IC_50_ values of the most active dose detected in AGS cells of these compounds were calculated and the effects of the compounds on DNA repair genes were studied by the RT-PCR method by RNA isolation and cDNA synthesis.

Compounds containing azomethine groups functioned as dioxygen carriers in catalytic reactions by conjugation with metals [26,27,28]. It has been reported that structurally modified azomethine group derivatives can be used as a bioactive substance in the cells of target tissues, thanks to their chemical reactivity [29,30]. It was noted that the hydroxyl groups containing the azomethines Co(III) complex showed anticancer activity [31,32]. In another study, the antiproliferative effects of azomethine derivatives on the MDA-MB-231 breast cancer cell line were investigated, and according to the results of the study, it was found to be dose-dependently decreased in breast cancer compared to the control group [33].

The results show that the numerical values obtained on the basis of the synthesized azo-azomethine data are in agreement with the mentioned experiments. The central role of all errors in DNA damage in the pathogenesis of cancer is unclear; however, these errors are of great importance in the progression and treatment of the disease.

In the studies, the overexpression of DNA repair genes has been reported to be associated with chemo- and radio-resistance in various tumors [34], and with the metastasis of tumors [35,36]. For this reason, the loss of DNA repair function at the beginning of tumor formation, reactivation, and the function of the genes involved here are of great importance in the progression of the disease [37,38]. In our study, we investigated the expression levels of compounds containing the azomethine group applied to gastric cancer cells on DNA repair genes. Ataxia telangiectasia and Rad3-associated (ATR), viz., a serine/threonine kinase, is an important regulator of genomic integrity that controls DNA replication stability, cell cycle checkpoints, and DNA repair [39]. ATR is activated in response to the DNA damage induced by ionizing radiation (IR) or anticancer drugs [40]. In the ATR-CHEK1 pathway, it has been reported that the activity and expression of ataxia telangiectasia (AT) cells, which do not have a functional ATM protein, are higher than normal cells [41]. In a study, high ATR-CHEK1 activity was detected in oral squamous cell carcinomas (OSCCs), and it was concluded that OSCC cells were protected from mitotic proliferation by enhancing the G2 phase of the cell [42]. Contrary to the studies mentioned in our current study, the *ATR* gene expression level was found to be lower in AGS gastric cancer cells compared to the control group. This suggests that low *ATR* expression may be associated with the loss of the G1 checkpoint as a result of chromosomal instability.

*Excision repair cross-complement 1 (ERCC1)* encodes a protein involved in NER and the interchain cross-link (ICL) repair of DNA and interacts with ERCC4 to form an endonuclease that cuts DNA for subsequent repair. ERCC1 increases the activity of the ERCC4 protein and provides stabilization [43,44]. It has been reported in studies that ERCC1 expression level differences cause platinum resistance in cell lines in ovarian, cervical, testicular, bladder, and non-small-cell (NSCLC) lung cancers [45]. In another study, it was noted that resected NSCLC patients with high *ERCC1* expressions had better survival rates compared to patients with low *ERCC1* expressions. In this study, it is assumed that the DNA repair mechanism contributes to the malignant potential of the tumor and that it may reduce the accumulation of thought genetic abnormalities and thus the risk of recurrence after definitive treatment [46]. In our current study, however, it was found that *ERCC1* gene expression increased in AGS gastric cancer cells in compound **3**, decreased in compound **2**, and remained unchanged in compound **1**. When the results are evaluated, it is reported that compound **3** has a good prognosis among gastric cancer in correlation with the studies employing high *ERCC1* gene expressions. In this case, the high expression of *ERCC1* is associated with the mechanism that influences tumor behavior, with the ability of this gene to repair DNA damage in gastric cancer cells. The low expression of compound **2** in *ERCC1* in AGS gastric cancer cells shows that it significantly reduces cellular viability, which correlates with a decreased DNA repair capacity. No significant difference was found between gastric cancer cells and *ERCC1* expression in compound **1**.

*The topoisomerase IIα gene (TOP2A)* encodes the enzyme topoisomerase IIa (topo IIa), which catalyzes the unwinding and recombination of double-stranded DNA. Type-II topoisomerases are the basic enzymes that break the double strand of the DNA backbone and convert the forms of DNA to each other, and they have important functions in a series of reactions, such as DNA replication, transcription, chromosome structure, condensation, and separation [47]. The protein levels of *TOP2A* in normal and cancerous cells vary during the cell cycle [48]. *TOP2A* expression in breast tumors has been reported to be associated with Ki-67 expression [49]. In vitro studies on various types of cancer have reported that sensitivity to TOP2A inhibitors depends on changes in the expression level of this gene, due to the fact that cells that are expressed at low levels show less sensitivity to drugs than those that are overexpressed [50]. In a study, it was shown that prostate cancer with a high *TOP2A* expression was associated with a lower survival rate [51]. In the present study, it was determined that azomethine group compounds in AGS gastric cancer, contrary to the mentioned studies, significantly reduced *TOP2A* gene expression levels and were associated with a good prognosis for gastric cancer. These results suggest that cells with a low *TOP2A* gene expression in gastric cancer are associated with low sensitivity to these synthesized compounds.

*The Atp-Binding Cassette (ABCB1/MDR)* gene encodes the transmembrane transporter P-glycoprotein that pumps various xenobiotic compounds out of the cell [52]. ABCB1/P-glycoprotein transports substrates from enterocytes into the intestinal lumen, thereby restricting the exposure of enterocytes to substrates of *ABCB1*, which is expressed in the plasma membranes of various cells and organs, including the blood–brain barrier (BBB) endothelium [53]. *ABCB1* gene expressions in solid tumors have been reported in the studies [54,55,56]. *ABCB1* protein levels were observed to be lower in colorectal cancer tissue compared to normal tissue in the immunohistochemistry analysis performed on 51 cancer patients [55]. In addition, it was reported that *ABCB1* mRNA and protein levels were lower in renal cell carcinoma tissues than in the normal cortex in 82 nephrectomized cancer patients [54] (p. 11). In accordance with the experiments conducted in our study, the *ABCB1* gene expressions of the compounds applied to gastric cancer cells were found to be significantly low. These results suggest that low ABCB1 levels increase intracellular exposure to carcinogenic ABCB1 substrates, thereby promoting gastric cancer.

In the study, these newly synthesized compounds were also evaluated biochemically in terms of oxidative stress (GSH), detoxification (GST), antioxidant (CAT), and cell survival (LDH). High GSH levels have been reported in various cancer types, such as breast, ovarian, lung, and head and neck cancers [57]. GSH has an important role in tumor initiation and the proliferation of increased cellular glutathione levels in tumors with many physiological functions [58,59]. In the current study, a 38% reduction in GSH levels was detected in the newly synthesized compound **2** applied to AGS gastric cancer cells. This indicates its antiproliferative effect on gastric cancer cells. It was noted that a decrease in the level of GSH could be harmful to cancer cells and potentially increase the effectiveness of chemotherapy and ionizing radiation [60,61,62]. GST levels vary in tissues and cells, and therefore each organ has different GST profiles. It was reported that while GSTA1 levels were increased in the kidney, liver, and testes, GSTP1 levels were higher in extrahepatic tissues [63,64]. It is also determined that GSTs are associated with carcinogenesis and drug resistance, and thus may be a biomarker for cancer [65]. In the current study, a 64% reduction was detected in compound **1** in AGS gastric cancer cell GST levels. Contrary to the aforementioned studies, it was noted that the low GST levels of this compound containing the azomethine group were associated with a good prognosis in gastric cancer.

CAT is the enzyme that induces the death of cancer cells by suppressing the anti-carcinogenic signals of increased reactive oxygen species and reducing oxidative stress [66,67,68]. In a study, it was reported that CAT levels were high in acute myeloid leukemia (AML) [69]. In our study, compounds applied to gastric cancer decreased CAT levels. This result suggests that intracellular H_2_O_2_ levels may increase and cancer may continue. LDH is the method used to detect anticancer activity and to determine the activity of cytoplasmic enzymes released from damaged cells. High-serum LDH levels are associated with various types of cancer, including solid tumors, such as pancreatic, prostate, and breast cancers, and hematological malignancies [70]. High-serum LDH levels are reported to be associated with poor prognosis for many types of cancer [71,72,73]. In our study, a slight increase in the LDH levels of compounds containing the azomethine group applied to AGS gastric cancer cells was detected. Even an increase in a certain rate reports the anticancer effect of these compounds on gastric cancer cells.

## 5. Conclusions

In our studies, the effects of newly synthesized azomethines on AGS gastric cancer were investigated in vitro and in silico. As a result of the analyses performed with theoretical computational methods, it was observed that the quantum chemical parameters of compounds **1**–**3** and the docking results were compatible. The molecular insertion results reveal that synthesized compounds **1**–**3** exhibit binding energies of −6.979, −8.100, and −8.906 kcal/mol, respectively, against the Hippo pathway, which is responsible for suppressing tissue and tumorigenesis in gastric cancer. In addition, the contour diagrams of boundary molecular orbitals and MEP maps showed that the -OH and CH=N substituents in the compounds were sites of nucleophilic attack at the molecular level. It was observed that this situation was similar to the interaction between the functional groups of the ligand and the amino acid residues of the proteins. In our in vitro experimental study, it was shown that newly synthesized Schiff bases had an important anticancer effect on AGS cancer cells and played a role by regulating the expression of some genes involved in oxidative stress and DNA repair. As a result, our study is important in terms of supporting the experimental data with bioinformatics and molecular insertion analyses. At the same time, there is a need to examine different biochemical parameters in order to convert the study findings into treatment methods.

## Figures and Tables

**Table 1:** Synthesized azomethine group-containing compounds.

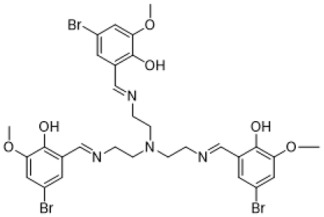	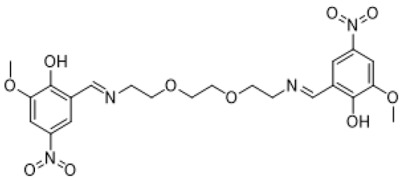
((nitrilotris(ethane-2,1-diyl))tris(azaneylylidene))	5,8-dioxa-2,11-diazadodeca-1,11-diene-tris(methaneylylidene))tris(4-bromo-2-methoxyphenol)
	1,12-diyl)bis(2-methoxy-4-nitrophenol)
**(Compound 1)**	**(Compound 2)**
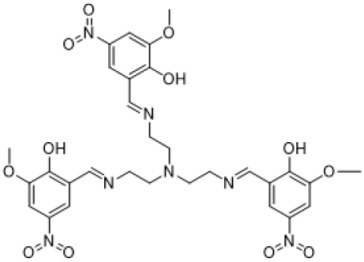	
((nitrilotris(ethane-2,1-diyl))tris(azaneylylidene))tris(methaneylylidene))tris(2-methoxy-4-nitrophenol)	
**(Compound 3)**	

**Table 2:** The molecular docking results.

	(1)-4LN0	(2)-4LN0	(3)-4LN0	Docetaxel-4LN0
DS *	−6.979	−8.100	−8.906	−3.999
E_vdW_ *	−19.563	−20.245	−20.841	−14.371
E_Coul_ *	−8.564	−10.525	−10.002	−7.001
E_Total_ *	−25.125	−27.005	−27.899	−24.514

* in kcal/mol.

**Table 3:** Predicted functional proteins associated with ATR, ERCC1, TOP2A, and ABCB1 (MDR1).

Proteins	Associated Proteins	Predicted Functional Proteins	Homology Score
ATR	TOPBP1	Serine/threonine protein kinase ATR	0.999
ATR	ATRIP	Serine/threonine protein kinase ATR	0.999
ATRIP	TOPBP1	Three-prime repair exonuclease 1	0.999
ERCC1	SLX4	DNA excision repair protein ERCC-1	0.999
ERCC1	XPA	DNA excision repair protein ERCC-1	0.999
ERCC1	ERCC4	DNA excision repair protein ERCC-1	0.999
ERCC4	XPA	DNA repair endonuclease XPF	0.997
ERCC4	SLX4	DNA repair endonuclease XPF	0.991
ATR	ERCC4	Serine/threonine protein kinase ATR	0.986
ATR	ERCC1	Serine/threonine protein kinase ATR	0.967
TOPBP1	ERCC4	Topoisomerase (dna) II-binding protein 1	0.957
ERCC1	TOPBP1	DNA excision repair protein ERCC-1	0.956
ATRIP	ERCC1	Three-prime repair exonuclease 1	0.941
ATRIP	ERCC4	Three-prime repair exonuclease 1	0.923
TOP2A	TOPBP1	DNA topoisomerase 2-alpha	0.875
XPA	ATR	DNA repair protein complementing XP-A cells	0.801
SLX4	TOPBP1	Structure-specific endonuclease subunit slx4	0.760
ATR	TOP2A	Serine/threonine protein kinase ATR	0.578
ABCB1	ERCC1	Multidrug-resistance protein 1	0.530
SLX4	ATR	Structure-specific endonuclease subunit slx4	0.526
ERCC1	TOP2A	DNA excision repair protein ERCC-1	0.509
XPA	SLX4	Topoisomerase (dna) ii-binding protein 1	0.508
TOPBP1	XPA	Topoisomerase (dna) ii-binding protein 1	0.469
ATRIP	XPA	DNA repair protein complementing XP-A cells	0.462
ATRIP	SLX4	DNA repair protein complementing XP-A cells	0.444

**Table 4:** RT-PCR results for all groups.

Genes	Groups	Mean CT	Fold-Change	*p*-Value
*ATR*	Compound **1**	27.29	0.09	0.001 *
	Compound **2**	28.89	0.01	
	Compound **3**	27.41	0.13	
	Control	27.71		
*TOP2A*	Compound **1**	25.58	0.47	0.001 *
	Compound **2**	26.37	0.10	
	Compound **3**	26.78	0.32	
	Control	28.36		
*ERCC1*	Compound **1**	25.64	0.96	0.001 *
	Compound **2**	26.08	0.26	
	Compound **3**	25.36	1.80	
	Control	29.45		
*ABCB1(MDR1)*	Compound **1**	28.31	0.04	0.001 *
	Compound **2**	29.56	0.01	
	Compound **3**	27.80	0.08	
	Control	27.45		
*GAPDH*	Compound **1**	25.27	1.00	
	Compound **2**	23.81	1.00	
	Compound **3**	25.90	1.00	
	Control	29.14	1.00	

* *p* < 0.05.

## Data Availability

Original data supporting the findings of this study are available. These data are available upon request from the corresponding author.
